# Soil and Leaf Nutrients Drivers on the Chemical Composition of the Essential Oil of *Siparuna muricata* (Ruiz & Pav.) A. DC. from Ecuador

**DOI:** 10.3390/molecules26102949

**Published:** 2021-05-15

**Authors:** Juan I. Burneo, Ángel Benítez, James Calva, Pablo Velastegui, Vladimir Morocho

**Affiliations:** 1 Departamento de Química y Ciencias Exactas, Universidad Técnica Particular de Loja (UTPL), Calle M. Champagnat s/n, Loja 1101608, Ecuador; jiburneo@utpl.edu.ec (J.I.B.); jwcalva@utpl.edu.ec (J.C.); pabloV-1@hotmail.com (P.V.); 2 Biodiversidad de Ecosistemas Tropicales-BIETROP, Herbario HUTPL, Departamento de Ciencias Biológicas, Universidad Técnica Particular de Loja (UTPL), Calle M. Champagnat s/n, Loja 1101608, Ecuador; arbenitez@utpl.edu.ec

**Keywords:** *Siparuna muricata*, guaiol, Cis-Cadina-1(6),4-diene, atractylone, multivariate analysis

## Abstract

Chemical compositions of plants are affected by the initial nutrient contents in the soil and climatic conditions; thus, we analyzed for the first time the effects of soil and leaf nutrients on the compositions of the essential oils (EOs) of *Siparuna muricata* in four different localities in Ecuador. EOs were obtained by hydrodistillation and analyzed by gas chromatography/mass spectrometry (GC/MS) and a gas chromatography/flame ionization detector (GC/FID). Enantiomeric distribution by GC/MS was determined, modifying the enantiomeric separation of β-pinene, limonene, δ-elemene, β-bourbonene, cis-cadina-1 (6), 4-diene and atractylone. A total of 44 compounds were identified. The most representative for L1 were guaiol, atractylone and 4-diene; for L2, cis-cadina-1(6),4-diene and myrcene; for L3, atractylone, myrcene and germacrene B; and finally, L4 germacrene B, myrcene and cis-cadina-1(6),4-diene. Correlations between soil- leaf chemical elements such as Al, Ca, Fe, Mg, Mn, N and Si in the different localities were significant with chemical composition of the essential oil of *Siparuna muricata*; however, correlations between soil and leaf K, P, and Na were not significant. Cluster and NMDS analysis showed high dissimilarity values of secondary metabolites between four localities related with changes in soil- leaf nutrients. Thus, the SIMPER routine revealed that not all secondary metabolites contribute equally to establishing the differences in the four localities, and the largest contributions are due to differences in guaiol, cis-cadina-1(6),4-diene, atractylone and germacrene. Our investigation showed for the first time the influences of altitude and soil- leaf chemical elements in the chemical composition of the EOs of *S. muricata*.

## 1. Introduction

Ecuadorian flora is recognized for its great diversity, with more than 16,000 vascular plant species [1], but in the last years this number has increased by 6%, exceeding the 17,548 species nowadays [2,3]. *Siparuna muricata* belongs to the *Siparuna* genus, which includes 235 species distributed in the tropical Americas [4], and around 40 species are found in Ecuador, mainly in the provinces of Loja, Zamora Chinchipe, Napo, Bolívar, Azuay, Chimborazo, Cañar, Imbabura and Tunguragua [5].

In this context, several studies shown that the Siparunaceae family has great economic, medicinal and phytochemical importance [6,7]. The fruits and leaves of certain species are used as traditional medicines of South and Central America to treat fever, cough, gastrointestinal diseases and rheumatism, and also this species is used as timber [8]. *Siparuna muricata* is an aromatic shrub or tree, also known as “limocillo” for its citrus smell, and thus alkaloids, flavonoids, tannins, sesquiterpene lactone, coumarins and cardiac glycosides are principal secondary metabolites [9]. Therefore, the *Siparuna* genus has good antimicrobial activity, regardless of the species; each one has a greater or lesser inhibitory activity for certain microorganisms [10,11].

The chemical, biochemical and pharmacological properties of certain plants can be defined by the characteristics of the chemical components present in their essential oils, which can be obtained from leaves, roots and stems [12]. Thus, elevation; precipitation; light; growing site; soil; anatomical, physiological and chemical variations between different parts of the plant, and from genetically-related factors might modify the qualitative and quantitative amounts of the volatile compounds in the EOs [13,14,15,16,17,18,19]. The composition and concentrations of the compounds of interest (active ingredients) may be linked to the place of origin of the plant species and to environmental and edaphological conditions [20]. They affect some processes associated with growth and development of the plants, even their ability to synthesize secondary metabolites [21]. The plants development and their production depend in part on the ability of the soil to supply and maintain an adequate amount of nutrients in the soil solution. Many of the nutrients that the plant requires for its development are found in the soil in varying amounts, and are sometimes insufficient for proper nutrition [22]. Following this pattern, several studies found that plant species growing under different environmental conditions (e.g., altitude and soil nutrients) show significant differences in the composition of the primary and secondary metabolite composition [23,24]. For instance, Zhao et al. [25] and Kumari et al. [26] for *Herpetospermum pedunculosum* and *Picrorhiza kurroa,* respectively, found correlations between secondary metabolites and altitude; similarly, Sampaio et al. [24] found that the distribution of the metabolites in *Tithonia diversifolia* was mainly affected by variation of some soil nutrients such as Ca, Mg, P, K and Cu.

Currently the most studies focus only on phytochemical analysis of *Siparuna* species [7,27,28,29,30,31] for instance, a phytochemical study in *Siparuna muricata* [9]. However, there is little information about the influences of soil and leaf chemical elements in relation to the composition of the essential oils. Thus, we determined for the first time the correlations between phytochemistry of *Siparuna muricata* and environmental and soil factors.

## 2. Results

### 2.1. Essential Oils (EOs) from Siparuna muricata Chemical Analysis

A total of 44 compounds were identified, representing 84.59% for L1, 92.50% for L2, 91.83% for L3, and 87.20% for L4. Guaiol, atractylone, β-copaen-4-α-ol, cis-cadina-1,4-diene, β-copaen-4-α-ol and germacrene B were most abundant chemical compounds in L1. For L2, they were cis-cadina-1(6),4-diene, myrcene, atractylone, β-Copaen-4-α-ol and germacrene B. For L3, they were atractylone, myrcene, germacrene B, β-copaen-4-α-ol, α-pinene and β-pinene. Finally in L4, germacrene B, myrcene, cis-cadina-1(6),4-diene, α-selinene and cis-guaia-3,9-dien-11-ol were most abundant chemical compounds (Table 1).

### 2.2. Enantiomeric Distribution

The enantiomeric distribution by GC/MS was determined in the MEGA DEX-DET-BETA stationary phase capillary column, by modifying the enantiomeric separation of β-pinene, limonene, δ-elemene, β-bourbonene, cis-cadina-1(6),4-diene and atractylone (Table 2). The enantiomeric distribution and the enantiomeric excess (e.e.) values of some monoterpenes were determined. None of the detected chiral compounds were present in their enantiomerically pure forms, however, δ-elemene was almost racemic, with only a small e.e. in favor of (−)-δ-elemene.

### 2.3. Antioxidant Capacity

The antioxidant capacity of the oil was evaluated via, spectrophotometric tests; in DPPH there were no significant reduction results, and in ABTS significantly results were found only for Chuquiribamba oil, with SC50 = 14.88 μg/mL (Table 3).

### 2.4. Physical Properties

Different physical properties of the essential oil were evaluated (Table 4). There were no differences between localities.

### 2.5. Leaf and Soil Chemical Analysis

Chemical composition of plants from four different localities (Table 5) showed that the nutrient with the highest content was phosphorus in a range between 133.84 and 201.47 ppm, and those with the lowest contents were manganese and iron in a ranges between 0.06 to 0.22 and 0.23 to 1.14 ppm, respectively.

Regarding the nitrogen content, we found that in Colaisaca (L4) there was a higher concentration of this element with a range between 9.81 to 12.05 ppm, showing significant differences compared to the other three study locations. The localities of Yangana (L2) and Celica (L3) had similar contents among themselves, and Chuquiribamba (L1) was the one with the lowest contents with the range of 3.7 to 8.8 ppm of nitrogen. Aluminum was another chemical element that had a high variability of results in each sampling area. Aluminum concentrations ranged from 1.11 to 15.99 ppm, with the highest value being found for L4. As for the sodium content, a great difference can be observed in the values of L1 with respect to the other three location, in L1 there are the highest values ranging between 4.73 to 4.84 ppm. Silicon has a similar tendency to sodium, since the highest values were found in samples from L1, reaching up to 7.23 ppm. For the elements of potassium, calcium and magnesium, there is also a great variability of results, for potassium from 0.78 to 1.99 ppm, for calcium, values between 0.78 to 4.05 ppm and for magnesium, values between 0.29 to 3.51 ppm.

On the other hand, aluminum is the chemical element that presented the highest concentrations in the soils of the studied localities, with a range between 9.96 to 54.78 ppm. Silicon was the second element with high values, observing that in this case, L1 was the one with the highest concentrations ranging from 22.49 to 23.12 ppm. Sodium also had high values but this was observed in L2 11.62 to 14.44 ppm (Table 6). The nitrogen and phosphorus had homogeneity of values in the four localities, in the case of nitrogen, values from 7.11 to 8.75 ppm were observed, and the phosphorus was between 7.01 to 8.21 ppm. On the other hand, manganese was the nutrient with the lowest concentrations between 0.05 to 0.01 ppm, in the same sense, potassium also has low values ranging from 0.15 to 0.73 ppm, however in both cases, observe the homogeneity of concentrations in the four study areas. Magnesium, on the other hand, presented values not greater than 5 ppm in the four locations, while calcium does not exceed 3 ppm, however in the latter case L4 was the one with the highest values compared to the other three locations in a range between 1.96 to 2.67 ppm.

### 2.6. Correlations between Soil and Plant Chemical Elements

The correlations between soil and leaf chemical elements as such as aluminum, calcium, iron, magnesium, manganese, nitrogen and silicium in the different localities were significant. On the other hand, correlations between soil and leaf potassium, phosphorus and sodium were not significant.

**Figure 1:**
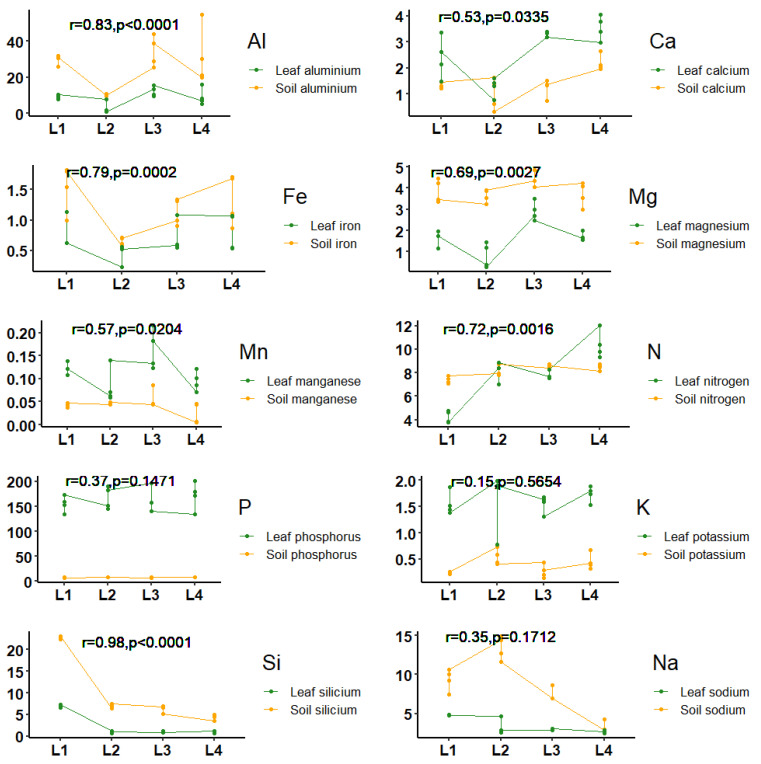
Correlations between soil and leaf chemical elements in the four studied localities.

Aluminum was the mineral that had the strongest correlation between soil and plant with respect to the rest of nutrient; that is, there was a linear relationship for the concentrations of nutrients between in the soil and plant (Figure 1).

### 2.7. Elements with Similar Chemical Properties in the Four Localities

**Figure 2:**
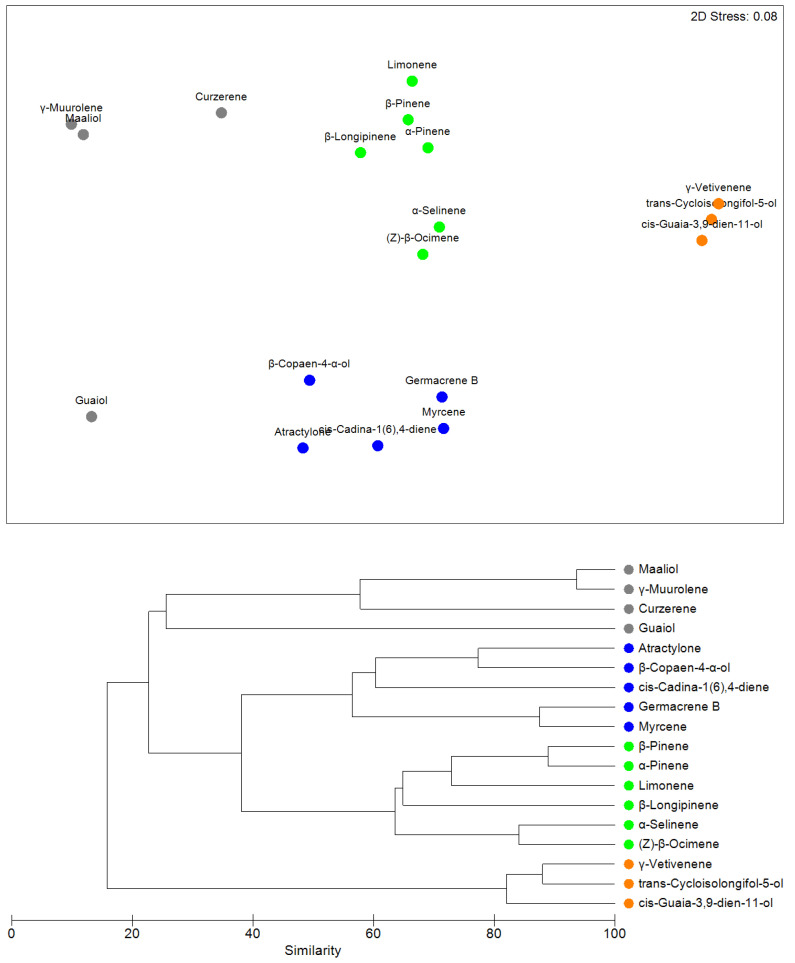
NMDS and Cluster analysis of secondary metabolites in the four localities. L1 Chuquiribamba (Grey circle); L2 Yangana (Green circle); L3 Celica (Blue circle), L4 Colaisaca (Orange circle).

In accordance with the NMDS and cluster results, chemical compounds of *Siparuna muricata* were grouped in a pattern related mainly to locality (Figure 2). For instance, between L1 and L4 show high dissimilarity of chemical compounds (67.62%). L3 and L4 had 45.69%, L1 and L3 with 45.47%, L2 and L4 with 42.85%, L1 and L2 with 32.32% and L2 and L3 with 28.36% of dissimilarity.

The SIMPER routine revealed that not all secondary metabolites contribute equally to establishing the differences in the four localities. We observed that the largest contributions are due to differences in guaiol, cis-cadina-1(6),4-diene, atractylone and germacrene B (Table 7).

## 3. Discussion

In our work we show that there are influences of soil and plant chemical elements in the composition of the essential oils of *Siparuna muricata*. We also observe that the nutrient content of the soil and the plant, as well as the chemical composition of the essential oil, are strongly influenced by the sampling location. Other studies found α-pinene (24.3%), β-pinene (21.7%) and myrcene (11.3%) in *Siparuna echinate* (Khunt) as major compounds [7], following these patterns, germacrene D (23.2% and α-pinene (7.0) were found to be major compounds in S. *macrotepala* [32]. A study of *S. schimpffii* Diels, which is used as an analgesic by the Shuar people of the Ecuadorian Amazon, specifically in the province of Morona Santiago, representative components were: germacrene D (35.34%), bicyclogermacrene (8.73%), γ-muurulene (7.04%), germacrene B (6.36%) and trans-cadina-1 (2), 4-diene (5.16%), which was similar with the chemical composition from our study, with the exception of germacrene D [33].

Several studies shown that physical, chemical and biological factors, both external and internal to the plant, can affect the quality and quantity of secondary metabolites [34,35,36]. For instance, Amzallag et al. [37], Vernin et al. [38] and Borges et al. [39] showed relations of secondary metabolites and environmental factors (e.g., elevation). In this context, the variability of the chemical composition of the essential oil of *S. muricata* found in our work could be explained by the different conditions of each locality, such as their altitudinal ranges, which are between 2184 to 2627 m a.s.l.

On the other hand, we showed influences of soil and plant chemical elements in the composition of the essential oils of *Siparuna muricata*. The quality and quantity of EOs of aromatic plants may be affected also by the physicochemical properties of soil [40]. Various chemical elements in soil found in rhizosphere of plants enter into the enzymes and affect the biochemical processes of plants [41]. The importance of soil in essential oil has also been reported in *Thymus hyemalis*, since individuals that develop in silty soils have a greater quantity of phenols and low molecular weight metabolites, compared to individuals found in clay soils [42]. In our work, a high variability of results in the content of the chemical elements both in the leaf of *S. muricata* and in the soils of the four sampling locations was found. According to Cruzatty and Vollmann [43], the content of nutrients in soils the related to its altitude gradient. The same authors state that the soils of Ecuador specifically in the sierra region (highlands), are little evolved and show high mineral contents, as is the case of our study areas, however, they have more acidic pH soils, because of stored organic matter and a high concentration of exchange aluminum.

The high soil and leaf aluminum contents are probably two of the characteristics that mark the most significant correlations between soil and leaf chemical elements of the *Siparuna muricata*. Thus, Lovkova et al. [44] mentioned that some soil ions (e.g., aluminum, cobalt, zinc and manganese), modulate the initial stages of biosynthesis of phenolic products in the plant. Hence, the soil chemistry can influence the phytochemical composition [45]. Soluble aluminum (Al^3+^) is the most limiting factor for plant growth in acid soils [46,47,48,49]; in the plant, it causes an alteration of the general metabolism, especially it inhibits root growth, which has as a consequence reduction in the intake of water and nutrients [50,51]. In addition, Al^3+^ increases the rigidity of the cell wall, interferes with the activity of several enzymes [52,53]. In such a way, the consequences of stress by Al^3+^ are presented at a biochemical and physiological levels, also influencing the essential oil compounds of plants.

Following this pattern, calcium, iron, magnesium, manganese, nitrogen and silicium also marked a significant correlation between soil and leaf chemical elements in the different localities. Silicium was also found in high concentrations in the plant and in the soil, both mainly in the locality L1 with values of up to 7.2 ppm for the plant and 23.12 ppm for the soil. Some research on silicon has shown that its presence in the plant as a constant element and in large proportion [54], as is the case with the results found in our work. In the case of nitrogen, values in the range from 7.11 to 8.75 ppm in soil were found, regarding the plant nitrogen content, we found that especially in L4 (Colaisaca) there was a higher concentration of this element with a range between 9.81 to 12.05 ppm, showing significant differences compared to the other three study locations. Studies on nitrogen mention that this element, as one of the important soil minerals, can alter the essential oil component through biosynthetic metabolic pathways [55]. In the study by Cruzatty and Vollmann [43], about the nitrogen fertilization methods affecting the essential oil and chemical composition of thyme, showed that nitrogen foliar application increased the vegetative yield, amount and percentage of essential oil and chemical compositions of thyme. These results agree with those found by Jabbari et al. [56], in which they mention that the highest yields of essential oils were obtained in the accessions of *L. origanoides* with the application of 100 kg/ha of nitrogen as urea.

On the other hand, soils elements such as P, K, Ca, Mg and Mn were those with low concentration values. This is consistent with the comments made by Duran et al. [57] in a physical-chemical analysis of soils that report concentrations even lower than those found in our study and who in turn mention that leaching, translocation and loss of the content of bases, cause an excessive accumulation of aluminum that generally decreases the assimilation of other mineral nutrients such as potassium, calcium and magnesium. Ross [58] reports in this work acidic soils with an exchangeable magnesium content below 40 ppm, in our study, we found even much lower magnesium values in soils with values not greater than 5 ppm in the four locations, this low amount of magnesium in the soil is also reflected in the low amount of this element in the S. *muricata* plant with values between 0.29 to 3.51 ppm. Both the exchangeable magnesium and the magnesium in the soil solution make up the magnesium available to the plant. Plants absorb magnesium exclusively from the soil solution which is replenished by magnesium from the exchangeable fraction. Magnesium deficiency in the plant leads to increased starch and dry matter content in older leaves, accompanied by deficiencies in carbohydrate supply to fruits, leaves, and particularly roots [59]. Who studied the effect of nutrition on the formation of palm oil shows higher oil/bunch ratios were produced through magnesium applications, so it can be that this element also influences the composition of the essential oils of plants.

## 4. Materials and Methods

### 4.1. Plant Material

Aerial parts of S. muricata were collected in four localities in the province of Loja south of Ecuador; L1: Chuquiribamba (79°16′55.4′′ W, 3°59′54.9′′ S at an altitude of 2545 m a.s.l.), L2: Yangana (79°9′54.39′′ W, 4°23′12.87′′ S at an altitude of 2256 m a.s.l.), L3: Celica (79°57′04.5′′ W, 04°05′45.8′′ at an altitude of 2184 m a.s.l) and L4: Colaisaca (79°41′14.5′′ W, 04°19′56.2′′ S at an altitude of 2627 m a.s.l) between the months of January to March 2018. The specimen was identified by the herbarium of the Universidad Técnica Particular de Loja (HUTPL) and deposited with voucher sample number HUTPL14257. The plant was collected under permission of the Ministry of Environment of Ecuador MAE-DNB-CM-2016-0048.

### 4.2. Essential Oil Distillation

The fresh samples were subjected by steam distillation in a Clevenger-type apparatus for 4 h. The essential oils were dried over anhydrous sodium sulphate and then stored in sealed vials, at −20 °C, ready for the gas chromatography/mass spectrometry (GC/MS) analysis.

GC/MS analysis was performed on an Agilent Technologies 6890 N gas chromatograph coupled to a mass spectrometer (Agilent Technologies 5973, Santa Clara, CA, USA) using a DB-5 MS (5% -phenyl-methylpolysiloxane, 30 m, 0.25 mm i.d., 0.25 μm film thickness; J & W Scientific, Folsom, CA, USA) capillary column. The oven temperature program was: 5 min at 60 °C, subsequently 3 °C/min up to 165 °C, then 15 °C/min up to 250 °C, held for 10 min. Injector temperature was 220°C, respectively. Helium (He) was used as the carrier gas, at a flow rate of 1 mL/min. Split ratio, 1:50; acquisition mass range, m/z 35–350. All mass spectra were acquired in electron-impact (EI) mode with an ionization voltage of 70 eV. Oil samples were diluted to 1:100 in methylene chloride (Fisher Scientific, Waltham, MA, USA, 99.9% purity), and the volume injected was 1 μL.

For the identification of the oil components was based in the comparison of peak retention time, retention index, and mass spectrum with that of authentic standards using a mixture of the homologous series of n-alkanes, from C9 to C20. The peak identification was based on the combination of linear retention indices and mass spectra with those reported in the WILEY275, NIST 17 and Adams [60] libraries. Quantification of essential oil components was performed using GC-FID, with the same method and instrumental configuration described for GC/MS by peak-area normalization by considering an equal response factor for the different chemical classes.

### 4.3. Enantioselective GC Analysis

The enantioselective GC analysis was performed on an Agilent 6890N, the instrument was equipped with a 2.3-diethyl-6-tert-butyldimethylsilyl-β-cyclodextrin enantioselective column, 25 m × 0.25 mm × film thickness 0.25 µm from Mega, Legnano, Italy, the following oven temperature program was used: 60 °C for 5 min, a gradient of 2 °C/min up to 220 °C, hold for 2 min. The elution order of the separated enantiomers was determined by injection of enantiomerically pure standards [61].

### 4.4. Antioxidant Capacity

*S. muricata* essential oil was assessed for its antioxidant capacity using DPPH and ABTS radical scavenging assays. The antioxidant capacity was expressed as SC_50_, as a measure of the scavenging potential of the essential oil. Butylated hydroxytoluene (BHT) and Trolox as positive control allowed us to compare this value.

### 4.5. Physical Propierties

The relativity density (d^20^) was determined using a pycnometer. The refractive index (n 20) was measured by an Abbe’s refractometer, manufactured by Boeco, Germany. The specific optical rotation ${\left[\propto \right]}_{20}^{D}$ was determined in a Hanon P 810 automatic polarimeter.

### 4.6. Plant and Soil Chemical Analysis

The samples of the plant correspond to leaves collected manually, subsequently dried in an oven with forced air circulation for 24 h and ground to obtain adequate homogeneity. Nitrogen was determined using the Kjeldahl method modified by Ramírez et al. (2013) [62] and its evaluation by UV-V spectrophotometer at a wavelength of 660 nm [63]. The phosphorus was extracted with acid mixture (concentrated HNO_3_ 65% and concentrated HCLO_4_ 70%) and its assessment using the ammonium vanadate molybdate method by ultra violet spectrophotometry visible at a wavelength of 430 nm. For the determination of K, Ca, Mg, Na, Mn, Al, Cu, Fe, Zn and Si in foliates, digestion with an acid mixture was carried out (concentrated NHO_3_ 65% and concentrated HCLO_4_ 70%), from the filtrate of the digestion were taken an aliquots by adding lanthanum oxide for the cases of K, Ca and Mg to minimize interference and determine them by Atomic Absorption Spectrophotometry [64]. For Na, Fe, Si, Mn, Cu, Zn and Al, the determination was made by direct reading in the filtrate obtained from the wet digestion and read in the Atomic Absorption Spectrophotometer.

The soil samples were collected with a hole at a depth of 20 cm, subsequently dried at room temperature and screened in 2 mm mesh. Nitrogen was determined using the Kjeldahl method modified Ramírez et al. [62] and its evaluation by UV-V spectrophotometer at a wavelength of 660 nm, while phosphorus was extracted with 50 mL of a solution containing 0.03 M NH4-F and 0.025 M HCl following the Bray-P method [63] and its evaluation by UV-V spectrophotometer at a wavelength of 882 nm. For the determination of K, Ca, Mg, Na, Mn, Al, Cu, Fe, Zn and Si in soils it was done with 0.1 M BaCl2 as an extractant and its measurement by Atomic Absorption Spectrophotometry at specific wavelengths of nutrients [65].

### 4.7. Data Analysis

Correlations between soil and leaf chemical elements of the essential oil of S. muricata were recorded in each locality using Pearson’s correlation coefficient. Non-metric multidimensional scaling (NMDS) was performed to detect patterns of similarity of chemical compound in relation to each locality. The NMDS was run using Sørensen (Bray–Curtis) as the distance measure, and the number of runs was 500. In addition, we used a cluster analysis (complete linkage) to classify all the secondary metabolites of the essential oil of *S. muricata* in each locality. Clustering was conducted using the unweighted pair group method with arithmetic averaging (UPGMA). To identify the secondary metabolites of the essential oil of S. muricata that contributed most to the dissimilarity between the four localities, we used the SIMPER statistical routine. All analyses were performed using R statistical software version 3.1.13 [66].

## 5. Conclusions

The variations in elevation, soil and leaf chemical elements significantly affect the compositions of leaf essential oils (EOs) of *Siparuna muricata* from different locations in the southern region of Ecuador. The soils of the four localities of our study revealed a high content of aluminum, and silicon and sodium. In the *S. muricata* we observed that the nutrient with the highest content was phosphorus, and those in least abundance were manganese and iron. Additionally, correlations between soil and leaf chemical elements as such as aluminum, calcium, iron, magnesium, manganese, nitrogen and silicon in the different localities were found.

The soil chemical elements and locations conditions affects different physiological and chemical processes of plants and therefore could affect significantly the yield and composition of EOs of *S. muricata*. Therefore, it is necessary to develop out long-term studies to understand the effects of the environmental conditions such as topography, clime and plant phenology to better understand the interactive relationships between locations, and plant and soil properties in the chemical components of the essential oil of *S. muricata*.

## Figures and Tables

**Table 1:** Chemical composition of the essential oil of *S. muricata* in DB-5ms.

N.	Compound	LRI	LRI_r_	%			
				L 1	L 2	L 3	L 4
1	α-Pinene	921	932	0.94 ± 0.01	2.31 ± 0.81	5.92 ± 1.21	2.11 ± 0.13
2	Camphene	936	946	0.10 ± 0.01	0.42 ± 0.04	0.25 ± 0.02	0.08 ± 0.01
3	β-Pinene	967	974	0.83 ± 0.01	2.37 ± 0.41	5.00 ± 1.21	0.95 ± 0.32
4	Myrcene	985	988	3.01 ± 0.43	16.57 ± 0.91	12.70 ± 0.98	17.95 ± 2.15
5	Limonene	1023	1024	0.77 ± 0.01	1.49 ± 0.65	2.54 ± 0.78	1.44 ± 0.91
6	(Z)-β-Ocimene	1032	1032	2.28 ± 0.01	5.77 ± 0.92	5.73 ± 0.65	5.17 ± 0.32
7	(E)-β-Ocimene	1042	1044	0.07 ± 0.01	0.86 ± 0.02	0.22 ± 0.01	0.26 ± 0.01
8	Isobornyl acetate	1277	1283	0.21 ± 0.01	--	--	--
9	δ-Elemene	1326	1335	0.28 ± 0.03	0.15 ± 0.01	0.46 ± 0.01	--
10	α-Cubebene	1337	1348	--	0.48 ± 0.04	--	0.28 ± 0.01
11	α-Ylangene	1362	1373	0.10 ± 0.01	0.23 ± 0.05	0.13 ± 0.01	0.14 ± 0.01
12	β-Bourbonene	1369	1387	--	0.30 ± 0.01	--	0.11 ± 0.01
13	Daucene	1376	1380	0.21 ± 0.01	0.26 ± 0.01	--	0.35 ± 0.01
14	β-Elemene	1379	1389	0.80 ± 0.01	0.63 ± 0.01	1.73 ± 0.78	1.62 ± 0.53
15	β-Longipinene	1403	1400	3.20 ± 0.52	1.91 ± 0.11	1.78 ± 0.11	2.47 ± 0.23
16	γ-Elemene	1418	1434	--	0.18 ± 0.01	0.25 ± 0.01	0.39 ± 0.04
17	α-Humulene	1438	1452	1.03 ± 0.05	0.47 ± 0.01	0.88 ± 0.05	0.65 ± 0.01
18	cis-Cadina-1(6),4-diene	1466	1461	13.31 ± 1.06	21.20 ± 2.01	1.98 ± 0.11	7.48 ± 0.41
19	β-Selinene	1472	1489	--	0.42 ± 0.01	1.00 ± 0.01	0.28 ± 0.01
20	γ-Gurjunene	1474	1475	1.81 ± 0.01	0.09 ± 0.01	0.18 ± 0.01	0.15 ± 0.01
21	γ-Muurolene	1477	1478	2.01 ± 0.01	--	--	--
22	γ-Himachalene	1479	1481	0.89 ± 0.03	0.98 ± 0.04	0.57 ± 0.01	0.69 ± 0.01
23	Curzerene	1482	1499	2.55 ± 0.11	--	2.72 ± 0.05	--
24	α-Amorphene	1486	1483	0.05 ± 0.01	0.12 ± 0.01	0.27 ± 0.01	--
25	α-Selinene	1489	1498	2.97 ± 0.06	2.75 ± 0.01	5.21 ± 0.74	6.76 ± 0.37
26	2-Tridecanone	1494	1495	--	--	0.30 ± 0.01	--
27	cis-β-Guaiene	1498	1492	--	0.90 ± 0.01	--	--
28	β-Vetispirene	1499	1493	--	--	--	1.26 ± 0.01
29	trans-Muurola-4(14),5-diene	1502	1493	0.62 ± 0.01	0.99 ± 0.07	0.57 ± 0.01	--
30	trans-Cycloisolongifol-5-ol	1504	1513	--	--	--	4.73 ± 0.01
31	δ-Amorphene	1505	1511	0.57 ± 0.01	--	0.67 ± 0.02	--
32	Bicyclogermacrene	1507	1500	0.66 ± 0.04	0.34 ± 0.01	1.04 ± 0.01	--
33	trans-Cadina-1,4-diene	1517	1533	0.07 ± 0.01	0.06 ± 0.01	--	--
34	γ-Vetivenene	1534	1531	--	--	--	3.72 ± 0.31
35	Germacrene B	1540	1559	4.09 ± 0.01	8.11 ± 0.56	12.18 ± 0.91	19.65 ± 0.64
36	Maaliol	1556	1566	2.28 ± 0.06	--	--	--
37	trans-Dauca-4(11),7-diene	1561	1556	--	--	--	1.25 ± 0.03
38	β-Copaen-4-α-ol	1589	1590	8.88 ± 0.01	9.81 ± 0.01	8.33 ± 0.01	--
39	Guaiol	1607	1600	14.61 ± 0.01	--	--	--
40	epi-α-Cadinol	1629	1638	0.40 ± 0.01	0.12 ± 0.01	0.17 ± 0.01	0.53 ± 0.01
41	Cubenol	1638	1645	0.88 ± 0.01		0.77 ± 0.01	--
42	α-Cadinol	1640	1652	0.92 ± 0.03	0.34 ± 0.01	0.60 ± 0.01	0.71 ± 0.05
43	cis-Guaia-3,9-dien-11-ol	1653	1648	--	--	--	6.03 ± 1.08
44	Atractylone	1673	1657	13.21 ± 1.01	11.91 ± 0.95	17.70 ± 0.98	--
	Monoterpene hydrocarbons			8.00	29.80	32.35	27.96
	Sesquiterpene hydrocarbons			32.65	40.52	28.90	47.24
	Oxygenated sesquiterpene			43.73	22.18	30.27	12.01
	Others			0.21	--	0.30	--
	Total			84.59	92.50	91.83	87.20

LRI: calculated linear retention indices obtained on DB-5ms column using a series of *n*-alkanes (C9–C24); LRIr: reference linear retention indices; %: relative percentage amount; L1: Chuquiribamba; L2: Yangana; L3: Celica; L4: Colaisaca.

**Table 2:** Enantioselective analysis of *Siparuna muricata* essential oil.

	L1		L2		L3		L4	
Enantiomers	Enantiomeric Distribution (%)	e.e. (%)	Enantiomeric Distribution (%)	e.e. (%)	Enantiomeric Distribution (%)	e.e. (%)	Enantiomeric Distribution (%)	e.e. (%)
(+)-β-Pinene	62.50	25.00	65.86	31.71	39.99	20.03	17.01	65.97
(−)-β-Pinene	37.50		34.15		60.02		82.99	
(+)-Limonene	22.85	54.29	20.25	59.50	25.43	49.13	12.17	75.67
(−)-Limonene	77.15		79.75		74.57		87.83	
(+)-δ-Elemene	48.76	2.47	49.53	0.94	49.03	1.93	--	--
(−)-δ-Elemene	51.24		50.47		50.97		--	
(+)-β-Bourbonene	--	--	71.74	43.49	--	--	--	--
(−)-β-Bourbonene	--		28.26		--		--	
(+)-cis-Cadina-1(6),4-diene	88.14	76.29	39.00	21.99	17.49	65.01	2.47	95.06
(−)-cis-Cadina-1(6),4-diene	11.86		61.00		82.51		97.53	
(+)-Atractylone	55.09	10.19	--	--	55.11	10.22	--	--
(−)-Atractylone	44.91		--		44.89		--	

Enatiomeric distribution EOs in the four localities, L1: Chuquiribamba; L2: Yangana; L3: Celica; L4: Colaisaca, e.e. = enantiomeric excess.

**Table 3:** Antioxidant capacities of *Siparuna muricata* EOs.

	DPPH		ABTS	
	SC50 (μg/mL) ± SD			
L1	3.41 ± 0.12	64.91 ± 1.71	463.80 ± 21.55	14.88 ± 0.04
L2	7.94 ± 0.51	63.83 ± 4,86	517.74 ± 34.72	58.54 ± 0.05
L3	4.2 ± 0.07	76.27 ± 1.03	16.61 ± 2.23	757.34 ± 0.10
L4	10.51 ± 0.03	88.14 ± 0.24	143.46 ± 25.01	161.36 ± 0.04

Antioxidant capacities in the four localities; L1: Chuquiribamba; L2: Yangana; L3: Celica; L4: Colaisaca.

**Table 4:** Physical properties of *Siparuna muricata* EOs.

	Yield (%)	Density (g/mL)	Refraction Index (ntD)	$\mathrm{Optical}\;\mathrm{Activity}\;\left[\alpha \right]\begin{array}{c} T\\ \lambda \end{array}$
L1	0.144 ± 0.03	0.933 ± 0.01	1.505 ± 0.01	+39,197 ± 0.91
L2	0.161 ± 0.04	0.914 ± 0.01	1.505 ± 0.01	−15,093 ± 0.85
L3	0.122 ± 0.02	0.917 ± 0.01	1.507 ± 0.02	−4180 ± 0.60
L4	0.111 ± 0.03	0.889 ± 0.02	1.501 ± 0.01	−38,928 ± 0.45

Physical properties in the four localities; L1: Chuquiribamba; L2: Yangana; L3: Celica; L4: Colaisaca.

**Table 5:** Plant chemical composition in the four localities.

		Ppm									
	Repetition	N	P	K	Ca	Mg	Mn	Na	Al	Si	Fe
**L 1**	1	4.64	133.84	1.88	3.37	1.76	0.11	4.79	9.38	6.59	0.63
	2	3.81	152.79	1.45	1.49	1.95	0.14	4.84	9.06	6.69	1.14
	3	4.75	159.11	1.53	2.13	1.18	0.11	4.73	7.89	6.57	1.13
	4	3.79	173.84	1.38	2.62	1.76	0.12	4.74	10.66	7.23	0.62
	$\bar{X}$	4.25 ± 0.52	154.89 ± 16.58	1.56 ± 0.22	2.40 ± 0.79	1.66 ± 0.33	0.12 ± 0.01	4.77 ± 0.05	9.25 ± 1.13	6.77 ± 0.31	0.88 ± 0.29
**L 2**	1	8.40	151.74	1.99	0.78	0.40	0.06	4.70	7.95	1.19	0.23
	2	8.87	190.95	0.78	1.42	1.18	0.06	2.89	1.44	0.80	0.57
	3	6.99	145.16	1.87	1.31	1.47	0.07	2.59	1.95	0.71	0.53
	4	8.87	182.26	1.91	1.60	0.29	0.14	2.86	1.11	0.96	0.52
	$\bar{X}$	8.28 ± 0.89	167.53 ± 22.47	1.64 ± 0.57	1.28 ± 0.35	0.84 ± 0.58	0.08 ± 0.04	3.26 ± 0.97	3.11 ± 3.25	0.91 ± 0.21	0.46 ± 0.15
**L 3**	1	7.69	196.74	1.63	3.19	2.69	0.13	2.92	13.47	0.91	0.59
	2	8.28	157.79	1.67	3.33	2.99	0.12	3.09	9.60	0.84	0.59
	3	7.58	141.47	1.59	3.40	3.51	0.22	2.92	10.25	1.25	0.55
	4	8.28	140.95	1.31	3.18	2.47	0.18	3.09	15.42	0.89	1.09
	$\bar{X}$	7.96 ± 0.38	159.24 ± 26.19	1.55 ± 0.17	3.28 ± 0.11	2.92 ± 0.45	0.16 ± 0.04	3.00 ± 010	12.19 ± 2.74	0.97 ± 0.19	0.71 ± 0.26
**L 4**	1	12.05	134.63	1.79	2.98	1.63	0.07	2.67	6.97	1.11	1.07
	2	9.81	179.89	1.54	3.79	1.99	0.10	2.85	8.46	0.92	0.55
	3	9.34	201.47	1.74	4.05	1.57	0.09	2.85	15.99	0.72	0.54
	4	10.40	172.00	1.89	3.40	1.68	0.12	2.49	5.25	1.21	1.06
	$\bar{X}$	10.40 ± 1.18	172.00 ± 27.85	1.74 ± 0.15	3.55 ± 0.47	1.72 ± 0.19	0.10 ± 0.02	2.72 ± 0.17	9.17 ± 4.73	0.99 ± 0.22	0.80 ± 0.30

Plant Chemical composition in the four localities, with his repetitions L1: Chuquiribamba; L2: Yangana; L3: Celica; L4: Colaisaca.

**Table 6:** Soil chemical composition in the four localities.

	Ppm										
	Repetition	N	P	K	Ca	Mg	Mn	Na	Al	Si	Fe
**L 1**	1	7.22	7.51	0.22	1.31	4.47	0.04	9.20	31.85	23.07	1.00
	2	7.11	7.41	0.22	1.23	4.24	0.04	10.10	30.88	22.49	1.54
	3	7.46	7.31	0.22	1.29	3.36	0.04	7.42	25.74	22.86	1.82
	4	7.76	7.21	0.26	1.47	3.45	0.05	10.65	31.20	23.12	1.80
	$\bar{X}$	7.39 ± 0.29	7.36 ± 0.13	0.23 ± 0.02	1.32 ± 0.10	3.88 ± 0.56	0.04 ± 0.00	9.34 ± 1.42	29.92 ± 2.81	22.89 ± 0.28	1.54 ± 0.38
**L 2**	1	7.93	7.51	0.73	1.62	3.25	0.04	14.44	10.16	6.43	0.58
	2	7.93	8.51	0.58	1.40	3.54	0.04	12.78	9.67	6.68	0.61
	3	7.81	7.51	0.44	0.63	3.88	0.04	14.93	10.84	7.38	0.71
	4	8.75	8.21	0.41	0.33	3.92	0.05	11.62	9.93	7.41	0.70
	$\bar{X}$	8.11 ± 0.43	7.94 ± 0.51	0.54 ± 0.15	1.00 ± 0.61	3.65 ± 0.31	0.05 ± 0.00	13.44 ± 1.53	10.15 ± 0.50	6.98 ± 0.50	0.65 ± 0.07
**L 3**	1	8.40	7.01	0.44	1.53	4.33	0.04	6.98	25.55	6.69	0.99
	2	8.28	7.21	0.20	0.75	4.86	0.05	6.98	29.13	6.60	1.31
	3	8.75	7.81	0.15	1.35	4.85	0.09	8.62	44.13	6.89	0.90
	4	8.64	7.51	0.29	1.37	4.04	0.04	6.98	38.87	5.10	1.33
	$\bar{X}$	8.52 ± 0.21	7.39 ± 0.35	0.27 ± 0.13	1.25 ± 0.34	4.52 ± 0.40	0.05 ± 0.02	7.39 ± 0.82	34.42 ± 8.58	6.32 ± 0.82	1.14 ± 0.22
**L 4**	1	8.16	7.41	0.43	1.96	4.24	0.01	2.87	19.82	3.58	1.69
	2	8.75	7.81	0.39	2.04	4.08	0.05	4.23	30.29	4.47	0.87
	3	8.52	8.11	0.32	2.67	3.54	0.04	2.63	54.78	4.90	1.11
	4	8.48	7.78	0.67	2.11	2.99	0.01	2.92	21.08	4.90	1.71
	$\bar{X}$	8.48 ± 0.24	7.78 ± 0.29	0.45 ± 0.15	2.19 ± 0.32	3.71 ± 0.57	0.03 ± 0.02	3.16 ± 0.72	31.49 ± 16.21	4.46 ± 0.62	1.34 ± 0.42

Soil Chemical composition in the four localities, with repetitions L1: Chuquiribamba; L2: Yangana; L3: Celica; L4: Colaisaca.

**Table 7:** Results of the SIMPER analyses.

Secondary Metabolites																		
	CA			CA			CA			CA			CA			CA		
	L1	L2	CD	L1	L3	CD	L1	L4	CD	L2	L3	CD	L2	L2	CD	L3	L4	CD
α-Pinene	0.94	2.31	2.37	0.94	5.92	6.99				2.31	5.92	7.67				5.92	2.1	5.2
β-Pinene	0.83	2.37	2.66	0.83	5	5.85				2.37	5	5.58				5	1	5.53
Myrcene	3.01	16.57	23.47	3.01	12.7	13.6	3.01	18	14.4	16.57	12.7	8.22				12.7	18	7.17
(Z)-β-Ocimene	2.28	5.77	6.05	2.28	5.73	4.85	2.28	5.17	2.79									
cis-Cadina-1(6),4-diene	13.31	21.2	13.66	13.3	1.98	15.9	13.3	7.48	5.62	21.2	1.98	40.82	21.2	7.48	19.7	1.98	7.5	7.51
γ-Muurolene	2.01	0	3.48															
Curzerene	2.55	0	4.42							0	2.72	5.78						
α-Selinene				2.97	5.21	3.15	2.97	6.76	3.65	2.75	5.21	5.23	2.75	6.76	5.75			
trans-Cycloisolongifol-5-ol							0	4.73	4.56				0	4.73	6.79	0	4.7	6.46
γ-Vetivenene							0	3.72	3.59				0	3.72	5.34	0	3.7	5.08
Germacrene B	4.09	8.11	6.96	4.09	12.18	11.4	4.09	19.7	15	8.11	12.2	8.65	8.11	19.7	16.6	12.2	20	10.2
Maaliol	2.28	0	3.95	2.28	0	3.2												
β-Copaen-4-α-ol							8.88	0	8.56				9.81	0	14.1	8.33	0	11.4
Guaiol	14.61	0	25.27	14.6	0	20.5	14.6	0	14.1									
cis-Guaia-3,9-dien-11-ol							0	6.03	5.81				0	6.03	8.65	0	6	8.24
Atractylone				13.2	17.7	6.3	13.2	0	12.7	11.91	17.7	12.3	11.9	0	17.1	17.7	0	24.2

CA: mean concentration (%); CD: contribution of each chemical compounds to the dissimilarity (%).
